# Frequent fire alters soil total phosphorus but does not affect phosphorus availability in a montane grassland

**DOI:** 10.1007/s10533-025-01304-w

**Published:** 2026-02-10

**Authors:** Nicola J. Findlay, Guy Thibaud, Alan D. Manson, Paul J. Gordijn, Max Rietkerk, Martin J. Wassen, Mariska te Beest

**Affiliations:** 1 Copernicus Institute of Sustainable Development, Utrecht University, Utrecht, The Netherlands; 2 Soil Fertility & Analytical Services, KwaZulu-Natal Department of Agriculture & Rural Development, Pietermaritzburg, South Africa; 3 School of Agricultural, Earth and Environmental Sciences, University of KwaZulu-Natal, Pietermaritzburg, South Africa; 4 South African Environmental Observation Network (SAEON), Grasslands, Forests and Wetlands Node, Pietermaritzburg, South Africa; 5 Biodiversity, Spatial Planning and Information, Ezemvelo KZN Wildlife, Pietermaritzburg, South Africa; 6 Centre for African Conservation Ecology, Nelson Mandela University, Port Elizabeth, South Africa

**Keywords:** Fire frequency, Soil phosphorus fractions, Montane grassland soil, Plant-available phosphorus, Andosols

## Abstract

Phosphorus (P) is often a limiting nutrient in highly weathered soils. Fire is a major driver of nutrient redistribution and can temporarily increase the pool of plant-available P in P-limited ecosystems. Yet, the long-term effects of frequent fire on soil P in montane grasslands remain poorly understood. We investigated how fire regime influences soil P pools using data from a long-term fire experiment in the South African Drakensberg. Total soil P, moderately labile organic and inorganic P and plant-available P were measured across five prescribed fire regimes varying in frequency (annual, biennial or infrequent) and season of burn (autumn or spring). We hypothesised that frequent fire would not alter total P in the topsoil, but expected it would increase inorganic P and plant-available P. Infrequent and biennial burns had little effect on total P; however, total P was significantly higher under annual spring burns than the other treatments, particularly the infrequent burns and annual or biennial autumn burns. In contrast, plant-available P did not respond to any fire treatment. Frequent spring burns generally increased organic P relative to inorganic P, indicating a shift in the composition of soil P pools with fire frequency and season. Overall, despite changes in topsoil total and organic P, plant-available P remained constrained, reflecting a bottleneck in the P cycle likely driven by the high P-retention capacity of these acidic Andosols. These findings highlight the complex and sometimes counterintuitive effects of fire on nutrient dynamics in montane grasslands.

**Supplementary Information:**

The online version contains supplementary material available at 10.1007/s10533-025-01304-w.

## Introduction

Phosphorus (P) is an essential element that is often limiting to primary production and strongly influences nutrient cycling in terrestrial ecosystems (Tiessen 2008). Unlike carbon (C) and nitrogen (N), which cycle through the atmosphere, P has no significant gaseous phase and is replenished through slow mineral weathering and biological recycling (Jahnke 1992). Consequently, disturbances that redistribute or deplete soil P pools can have long-term effects on ecosystem-scale biogeochemical processes and properties (Vitousek et al. 2010; Butler et al. 2017; Wassen et al. 2021). Among these disturbances, fire is a major driver of nutrient redistribution (Liao et al. 2013; Zhou et al. 2025), with significant consequences for productivity and key ecosystem processes such as nutrient mineralisation, plant nutrient uptake and broader biogeochemical cycling. Anthropogenic climate change is projected to intensify fire regimes worldwide by increasing atmospheric carbon dioxide (CO_2_) concentrations, elevating vapour pressure deficits, and altering rainfall and temperature patterns (Ainsworth & Long 2005; Taub 2010; Zhu et al. 2016; Rao et al. 2023). These shifts are expected to increase fuel loads and flammability (Clark 1988; Leys et al. 2018), leading to more frequent and severe fires with significant implications for nutrient cycling. Understanding how fire affects soil P dynamics is therefore increasingly important for predicting ecosystem responses under global environmental change.

Grasslands warrant particular attention in this context. Covering over 40% of the Earth’s terrestrial surface, they provide essential ecosystem services, supporting global food systems and water supplies, storing substantial belowground C, and contributing disproportionately to global biodiversity (Bengtsson et al. 2019). Despite their importance, grasslands are among the biomes most threatened by land-use conversion and climate change (Hoekstra et al. 2005). Nutrient limitation, particularly co-limitation by N and P, is a feature of many grasslands (Craine et al. 2008; Fay et al. 2015). Moreover, unlike forests, where biomass accumulates over decades, grassland productivity and resilience depend on rapid nutrient cycling within shallow, dynamic soil layers. Fire can play a defining role here by maintaining open structure, promoting primary production, and influencing nutrient cycling (Bond 2008; Staver et al. 2011; McLauchlan et al. 2020). However, most research on fire effects on soil P has focused on tree-dominated ecosystems, where nutrient cycling is concentrated beneath tree canopies (Pellegrini et al. 2015; Coetsee et al. 2010). Non-woody grasslands, particularly temperate montane grasslands, remain comparatively understudied (Butler et al. 2018; Cheng et al. 2025) and it is unclear whether patterns observed in wooded systems apply to them. This highlights an important knowledge gap, especially as grassland fire regimes are projected to intensify (Sayedi et al. 2024).

**Fig. 1:**
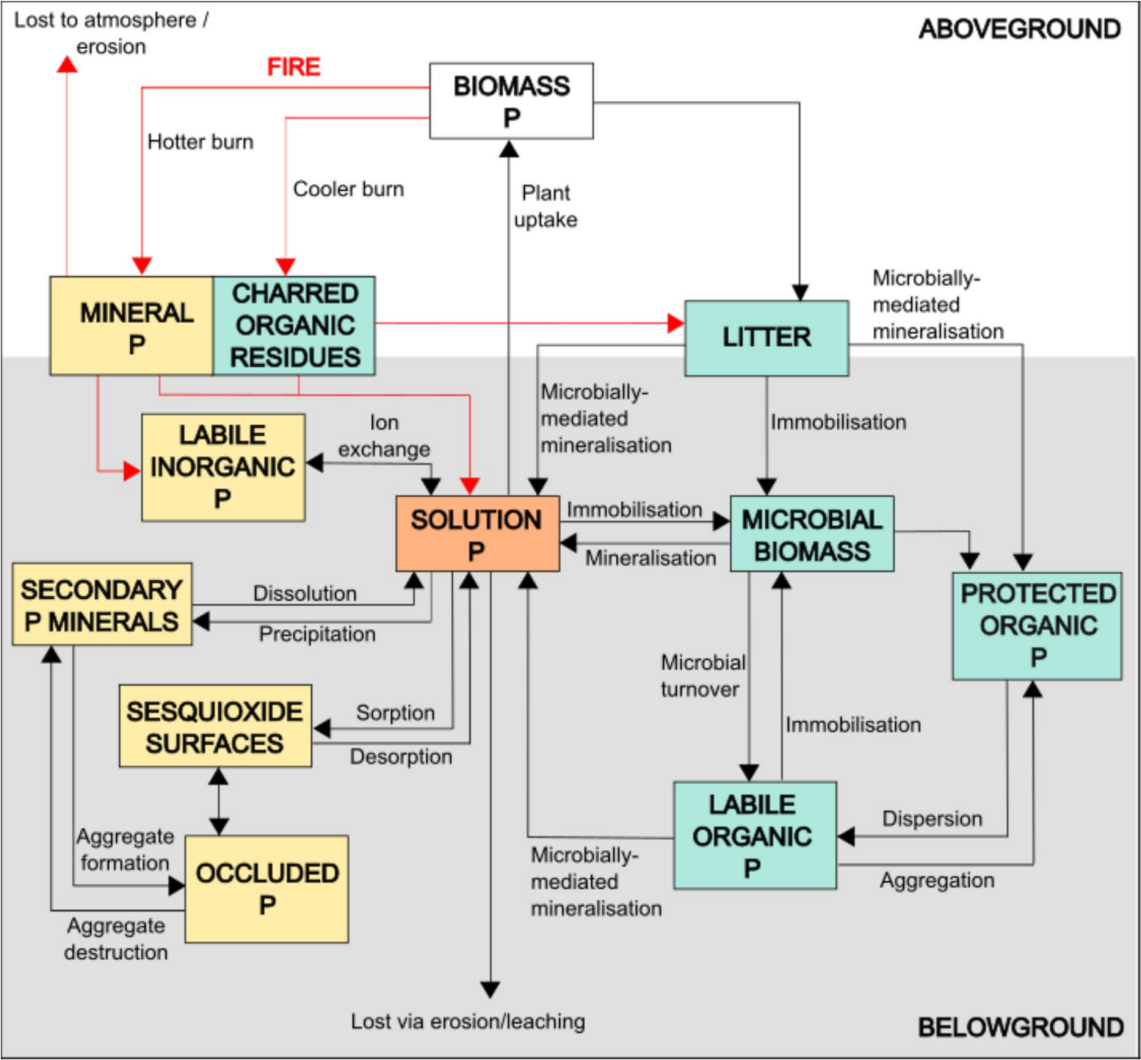
Soil P pools and the main mobilisation processes in the soil P cycle. Red flux lines represent processes expected to be directly affected by fire – such as combustion of plant biomass, ash deposition, and post-fire erosion – while black lines indicate fluxes not expected to be directly affected by fire. Hot fires lead to more complete combustion of organic material, increasing mineral P inputs to the labile inorganic P pool in the soil. Cooler burns, in contrast, leave some plant biomass as P-containing charred organic residues, which contribute to the litter layer. Both mineral P and charred organic residues ultimately contribute to the solution P pool. The diagram is divided into two main components: the organic P pool (represented by the pale blue boxes) and the inorganic P pool (represented by the yellow boxes). The central orange box represents P dissolved in the soil solution (plant-available P), which is readily accessible for plant uptake and microbial assimilation and consists predominantly of inorganic orthophosphate ions (PO_4_^3−^)

Quantifying fire-induced shifts in soil P requires distinguishing among pools that differ in bioavailability. Although sequential fractionation methods are widely used to separate P fractions, their ecological interpretation remains debated (Kruse et al. 2015; Weihrauch & Opp 2018). In soils, P primarily occurs as orthophosphate ions (PO_4_^3−^), which may be present in the soil solution as readily available inorganic P for plant or microbial uptake, or bound within organic matter (organic P, P_o_), or adsorbed to mineral surfaces (inorganic P, P_i_). These forms are distributed among pools that differ in solubility and accessibility (Weihrauch & Opp 2018), ranging from readily available “solution” and “labile” P to more stable “sorbed” and “occluded” fractions (Hedley et al. 1982) (Fig. 1). Understanding how fire alters the partitioning of P among these pools is essential for predicting its effects on short-term nutrient turnover and long-term soil fertility (Merino et al. 2019).

Fire can substantially modify ecosystem P dynamics, typically increasing P_i_ while reducing P_o_ pools (Romanyà et al. 1994; Butler et al. 2018; Deng et al. 2024; Souza-Alonso et al. 2024). In unburned systems, P_o_ mineralisation depends on microbial activity and the release of phosphatase enzymes from microbes and plant roots (McGill & Cole 1981; Ölinger et al. 1996). Fire-driven transformations bypass these biochemical pathways, rapidly changing nutrient availability. During combustion, most P in phytomass is retained within the system, remaining on the soil surface as P_i_ in ash or P_o_ in charred residues (Cofer et al. 1997; González-Pérez et al. 2004; Hartshorn et al. 2009). Even relatively low intensity fires (~ 150 °C) can induce pyromineralisation of P_o_ (García-Oliva et al. 2018), temporarily enhancing P bioavailability and producing a “P-rich signature” in post-fire soils (Schaller et al. 2015; Butler et al. 2018). Ash deposition can further modify soil chemistry by increasing P solubility in acidic soils through localised liming effects (Bodí et al. 2014). Fire-driven nutrient responses also depend on burn season (Findlay et al. 2022). In regions with pronounced seasonality, dry-season fires tend to be more intense, consuming more biomass and producing greater quantities of ash. However, these burns can leave bare soils exposed for months before post-fire rainfall stimulates vegetation recovery, increasing vulnerability to erosion. During this period, erosion of nutrient-rich ash can offset or even reverse nutrient gains (Resende et al. 2011; Nunes et al. 2018; Findlay et al. 2022).

In this study, we assess the effects of long-term, frequent fire on soil P fractions using the 39-year Brotherton fire experiment on a plateau in the temperate grasslands of the uKhahlamba-Drakensberg (hereafter Drakensberg) mountains, which form part of the eastern Great Escarpment in South Africa. These fire-dependent grasslands are renowned for their biodiversity and high levels of endemism. Biennial prescribed burns are implemented to sustain biodiversity and ecosystem services (Everson 1985; Morris et al. 2020). Extending across elevations of 1800–2500 m above sea level, the high-organic matter soils in the region are derived primarily from weathered basalt and many display the distinctive chemical properties of acidic alu-andic soils (FAO 2014; Kögel-Knabner & Amelung 2014; Findlay et al. 2022). Given the geomorphological setting, P cycling in the topsoils of the experimental site may approach a quasi-stable equilibrium in which surface P losses, driven by strong late-winter katabatic winds (Brown & Piper 1988), are offset by inputs from ash deposition and the vertical redistribution of P from deeper soil layers (Porder et al. 2007; Siebers et al. 2021).

The central aim of this study was to determine how prescribed burning influences the distribution of soil P across ecologically relevant pools in these montane grassland soils. We hypothesised that total topsoil P would not differ significantly between frequent and infrequent fire regimes, as fire-driven inputs and internal redistribution are expected to maintain overall P stocks. In contrast, we predicted fire to alter the distribution of P forms in the topsoil: specifically, that P_o_ would decline with repeated fire due to biomass loss, while P_i_ would increase owing to ash deposition and pyromineralisation. As a result of the expected increase in P_i_ in the topsoil, we also predicted that plant-available P would increase under frequent fire. Changes in deeper soil layers (5–15 cm) were expected to be minor, reflecting limited direct fire effects at depth.

## Materials and methods

**Fig. 2:**
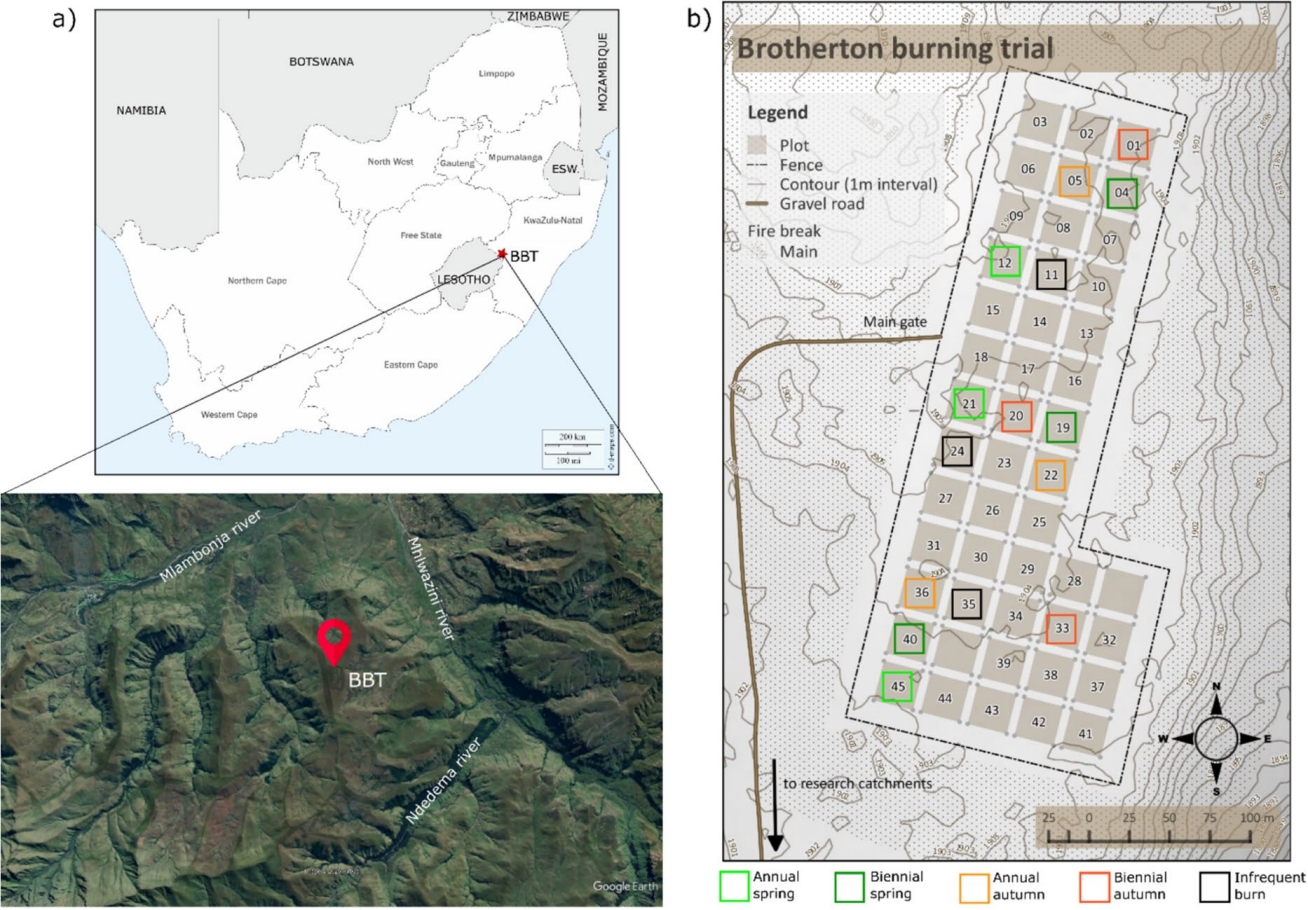
**a** Location map of the Brotherton burning trial (BBT) and **b** experimental plot layout showing the relative positions of selected burning treatments

A comprehensive overview of the site and experiment is given in Findlay et al. (2022). In brief, this study was conducted at the Brotherton burning trial (Fig. 2), which lies on the Brotherton ridge in the northern section of the uKhahlamba-Drakensberg Park in South Africa (28°58.2’S, 29°15.7’E; 1900 m a.s.l.). The humid temperate climate (Everson & Everson 2016) is characterised by moderate temperatures, with a long-term mean annual temperature (MAT) of 13.8 °C for the period 1949–1990 (Everson et al. 1998). Over the most recent decade (2013–2025), MAT has increased by 1.13 °C (Toucher 2024). The area receives an average of 1300 mm of annual precipitation (1949–1990; Everson et al. 1998), predominantly from summer thunderstorms with occasional winter snowfalls (Tyson et al. 1976). Precipitation over the past decade remains similar to the long-term average, with 1274 mm (2013–2025; Toucher 2024). The soils across the experiment can be classified according to USDA soil taxonomy as lithic Haplustands (Soil Survey Staff 2014); they are shallow, acidic andisols (pH_KCl_ range between 4.08 and 4.3 in the top 15 cm) that are derived from deeply-weathered Drakensberg basaltic lavas (King 1944; Findlay et al. 2022). Under the World Reference Base (WRB), these soils correspond to alu-andic Andosols (FAO 2014). Despite their limited depth (0.3 to 0.5 m), they support a substantial accumulation of organic matter (Manson et al. 2007). Soil classification did not differ across the study area, ensuring that observed fire effects are not confounded by underlying variation in soil taxonomy. The vegetation at the site is classified as uKhahlamba Basalt Grassland (Mucina & Rutherford 2006). While the C4 grass *Themeda triandra* dominates*,* the grasslands are home to a rich diversity of montane flora, including 188 flowering species that are endemic to the Drakensberg’s montane altitudinal belt (Carbutt 2019).

## Experimental design and data collection

The Brotherton burning trial is a long-term fire experiment that was initiated in 1980 to assess the impact of fire regime on species composition and fire behaviour in montane grasslands (Granger 1979, unpublished report; O’Connor et al. 2004). In this study we selected five fire treatments, each with three replications: annual burning in autumn or spring, biennial burning in autumn or spring, and fire exclusion. Since its inception, the Brotherton burning trial has experienced three unplanned fires in the winters of 2000, 2007 and 2016 (te Beest et al. 2021), which led to the classification of the fire exclusion treatment as an infrequent burn. Autumn burns are conducted in May, following the first frost, while spring burns take place in October after the first substantial rainfall.

**Fig. 3:**
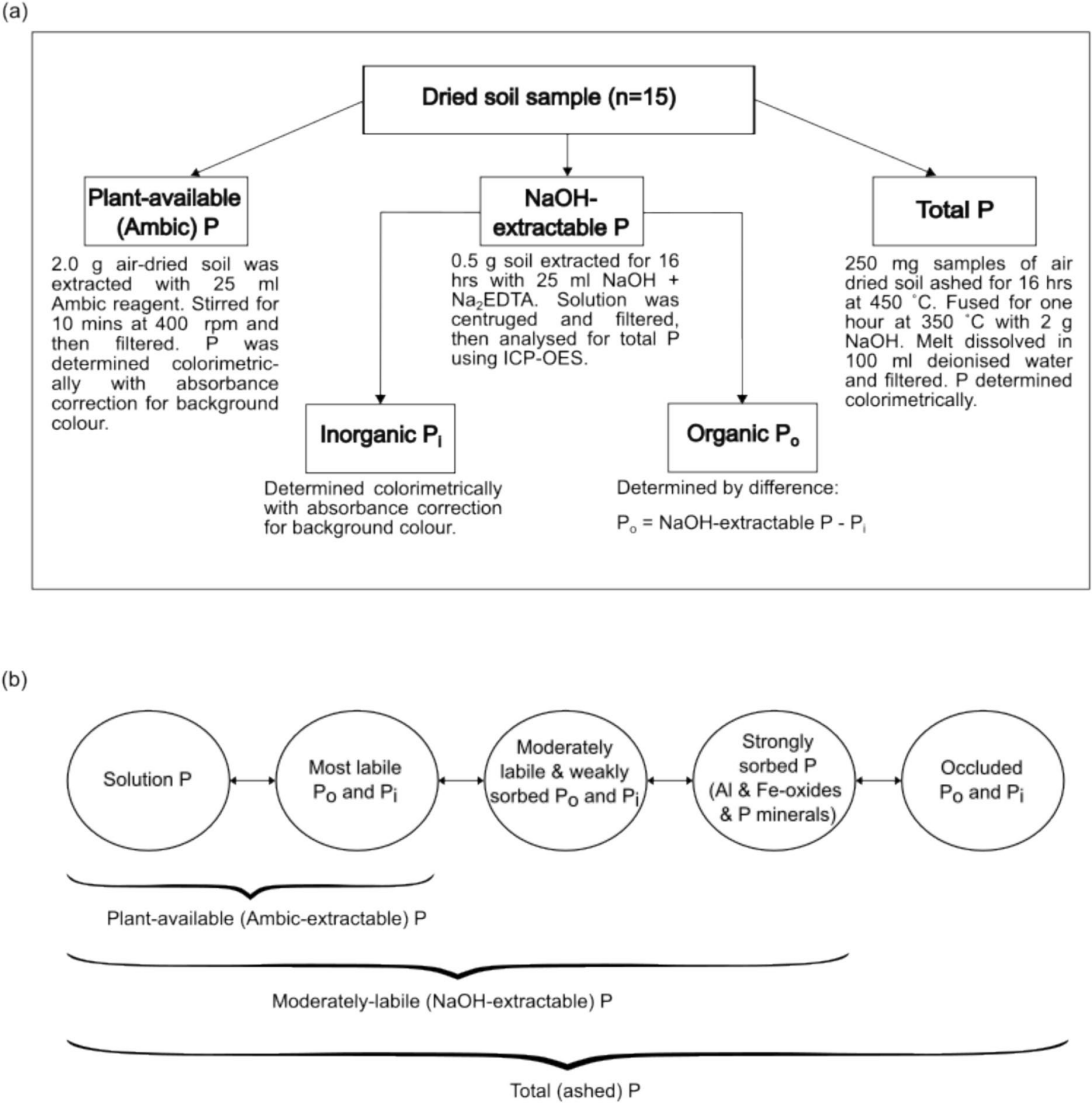
Schematic diagram of **a** sample preparation and analysis procedure and **b** a representation of the P fractions measured and how they add to certain P pools. Note that the pools increase in size from left to right in part **b** of the figure. Ambic-extractable P is a subset of the most labile inorganic P

Soil samples were taken in March 2019 at two depth intervals of 0–5 cm and 5–15 cm from the dedicated two-metre-wide sampling area that is located around the inner perimeter of each plot using a Beater auger (custom made by Hayden’s Engineering, Pietermaritzburg, KwaZulu-Natal, South Africa). Twenty discrete samples were collected for each depth interval per plot. These were combined into a composite sample for analysis, resulting in one sample per depth interval per plot (Fig. 3).

### Phosphorus analyses

Analyses were conducted on the air-dried composite samples, which were first gently crushed to pass through a 1 mm brass sieve (Advantech Manufacturing Inc., ATM 18BS8F, New Berlin, Wisconsin, USA) (Findlay et al. 2022). Total P was determined after dry ashing overnight at 450 °C, followed by fusion with sodium hydroxide (NaOH) for one hour at 350 °C in a high temperature muffle furnace (Ultra-Furn SA (Pty) Ltd., Model E15/30, Germiston, Gauteng, South Africa) (modified from Smith & Bain 1982). After dissolving in deionised water and filtering through Whatman Grade 1 qualitative filter paper (11 µm retention; Cytiva, Cat. No. 1001–150, Maidstone, Kent, United Kingdom), aliquots of the filtrate were analysed for P following the Murphy & Riley (1962) molybdenum blue procedure.

Total extractable P (hereafter NaOH-extractable P) was determined following the NaOH and disodium ethylenediaminetetraacetic acid (Na_2_EDTA) method of Bowman & Moir (1993) and analysed using Inductively Coupled Plasma Optical Emission Spectroscopy (ICP-OES, 5800 series, Agilent Technologies Inc., Santa Clara, CA, USA). The inorganic P fraction in the extracts was quantified by measuring molybdate-reactive P, also using the method of Murphy and Riley (1962). Background colour due to organic matter was corrected for by measuring the difference in absorbance of aliquots treated with or without ascorbic acid added to the molybdate reagent (O’Halloran & Cade-Menum 2008). Organic P was calculated by subtracting inorganic P from NaOH-extractable P.

Plant-available P was determined by extracting soil samples with an Ambic solution comprising 0.25 mol L^−1^ ammonium bicarbonate (NH_4_HCO_3_) + 0.01 mol L^−1^ ammonium fluoride (NH_4_F) + 0.01 mol L^−1^ Na_2_EDTA + Superfloc® N100 (0.05 g L^−1^), adjusted to pH 8.0 (van der Merwe et al. 1984; Manson et al. 2020). Background colour due to organic matter was corrected for as described above and the P concentration determined using the molybdenum blue procedure. To aid interpretation of the unexpected lack of response of plant-available P to fire regime, we conducted supplementary post hoc analyses of soil P retention capacity, following the methods described by Blakemore et al. (1987). Approximately 5 g of air-dried, sieved soil (1 mm) was equilibrated with 25 mL of a 1 mg P mL^−1^ potassium dihydrogen phosphate (KH_2_PO_4_) solution buffered at pH 4.6 with 0.2 mol L^−1^ anhydrous sodium acetate (CH_3_COONa) and 0.2 mol L^−1^ glacial acetic acid (CH_3_COOH) for 16 h with shaking at room temperature. After centrifugation, the phosphate remaining in solution was measured colorimetrically at an absorbance of 420 nm using a nitric vanadomolybdate reagent. Phosphorus retention was calculated as the difference between initial and final phosphate concentrations, expressed as a percentage of phosphate retained by the soil.

All analytical reagent grade chemicals were purchased from Sigma-Aldrich, South Africa.

### Statistical analysis

We tested for significant effects of fire regime on each soil P fraction, for each depth, using a one-factor analysis of variance (ANOVA). Due to low sample sizes and the resulting unbalanced design across fire treatment combinations, fire frequency and fire season were combined into a single categorical factor (“fire regime”) for the purposes of analysis. Fisher’s protected Least Significant Difference (LSD) was used to compare treatment differences at a significance level of *α* = 0.05.

To assess whether differences in nutrient concentrations were influenced by the time elapsed since the most recent fire (TSLB), we ran supplementary ANOVA models that included TSLB as a continuous covariate. These analyses showed that TSLB did not significantly explain variation in soil P (*P* > 0.05), and therefore it was not retained in the final models presented in the Results.

All statistical analyses were performed using GENSTAT 18 (VSN International 2015) and figures were built with R Studio v4.3.3 (Posit team 2024) using the *ggplot2* (Wickham 2016) and *dplyr* (Wickham et al. 2023) packages.

## Results

**Fig. 4:**
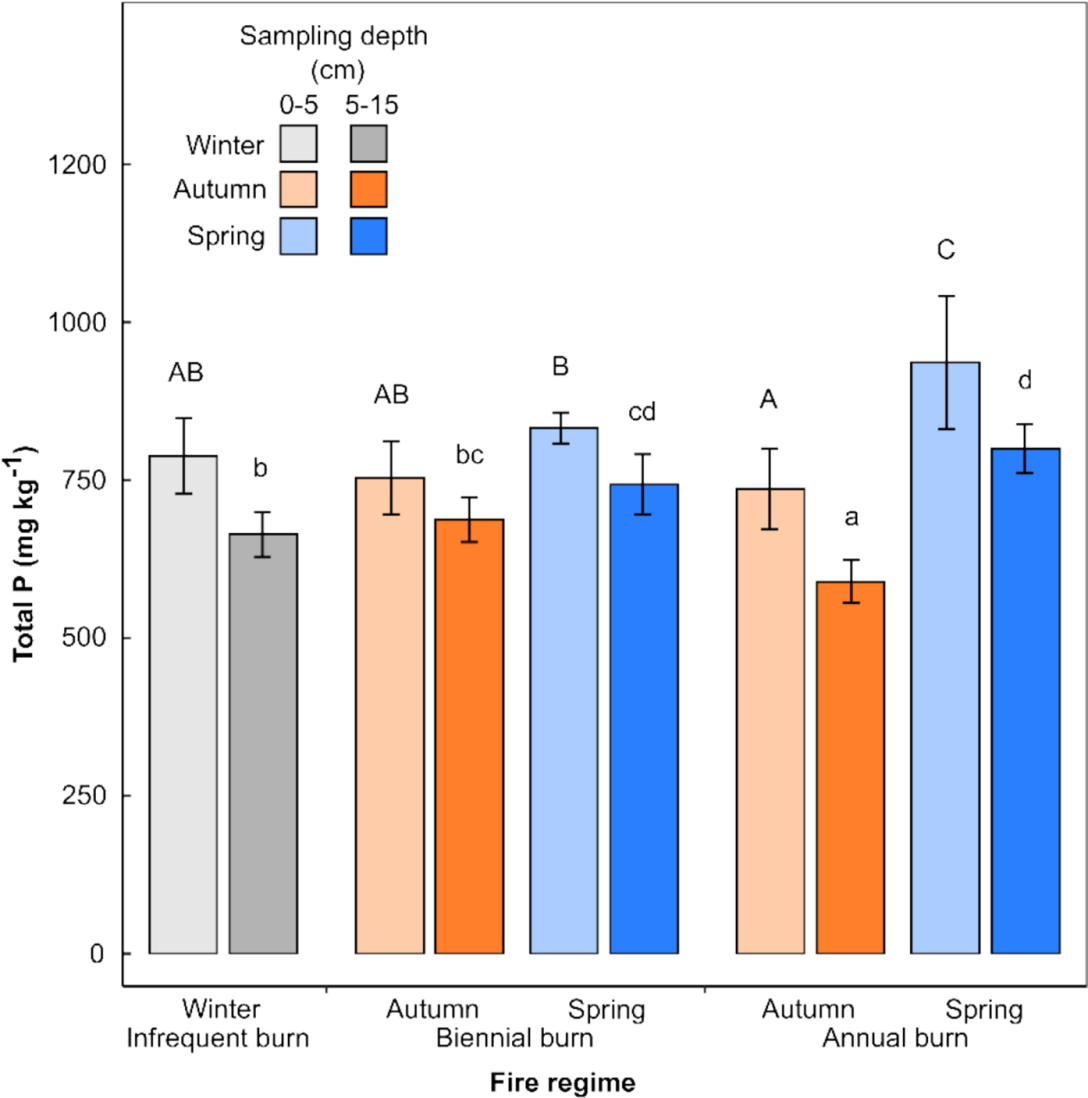
Average total soil P (± 1 SD, *n* = 3) in the 0–5 cm and 5–15 cm depths, determined by ashing and fusion with NaOH. Distinct letters signify significant differences between the treatments (upper and lower case representing the 0–5 cm and 5–15 cm sampling depths, respectively) determined by post hoc testing (Fisher’s protected LSD), at *α* = 0.05. There were significant differences in P concentration with depth (*P* = 0.008), hence a separate ANOVA was conducted for each sampling depth

Total soil P ranged from 687 to 1052 mg kg^−1^ in the top 0–5 cm of soil, and from 561 to 844 mg kg^−1^ in the 5–15 cm depth. Overall, total soil P decreased significantly with depth (*P* < 0.001, lsd_0.05_ = 64.4), from an average of 809 mg kg^−1^ in the 0–5 cm depth to 697 mg kg^−1^ in the 5–15 cm depth. However, no significant interaction was found between treatment and depth (Fig. 4).

Fire regime had a significant effect on total soil P at both the 0–5 cm (*P* = 0.008, lsd_0.05_ = 95.6) and 5–15 cm depths (*P* = 0.002, lsd_0.05_ = 73.7). Total P was highest in the annual spring burn treatment, with values of 936 mg kg^−1^ in the 0–5 cm depth and 800 mg kg^−1^ in the 5–15 cm depth. In contrast, total P was lowest in the annual autumn (736 and 590 mg kg^−1^) and infrequent burn treatments (789 and 664 mg kg^−1^). The differences between treatments were more pronounced in the 5–15 cm depth.

**Fig. 5:**
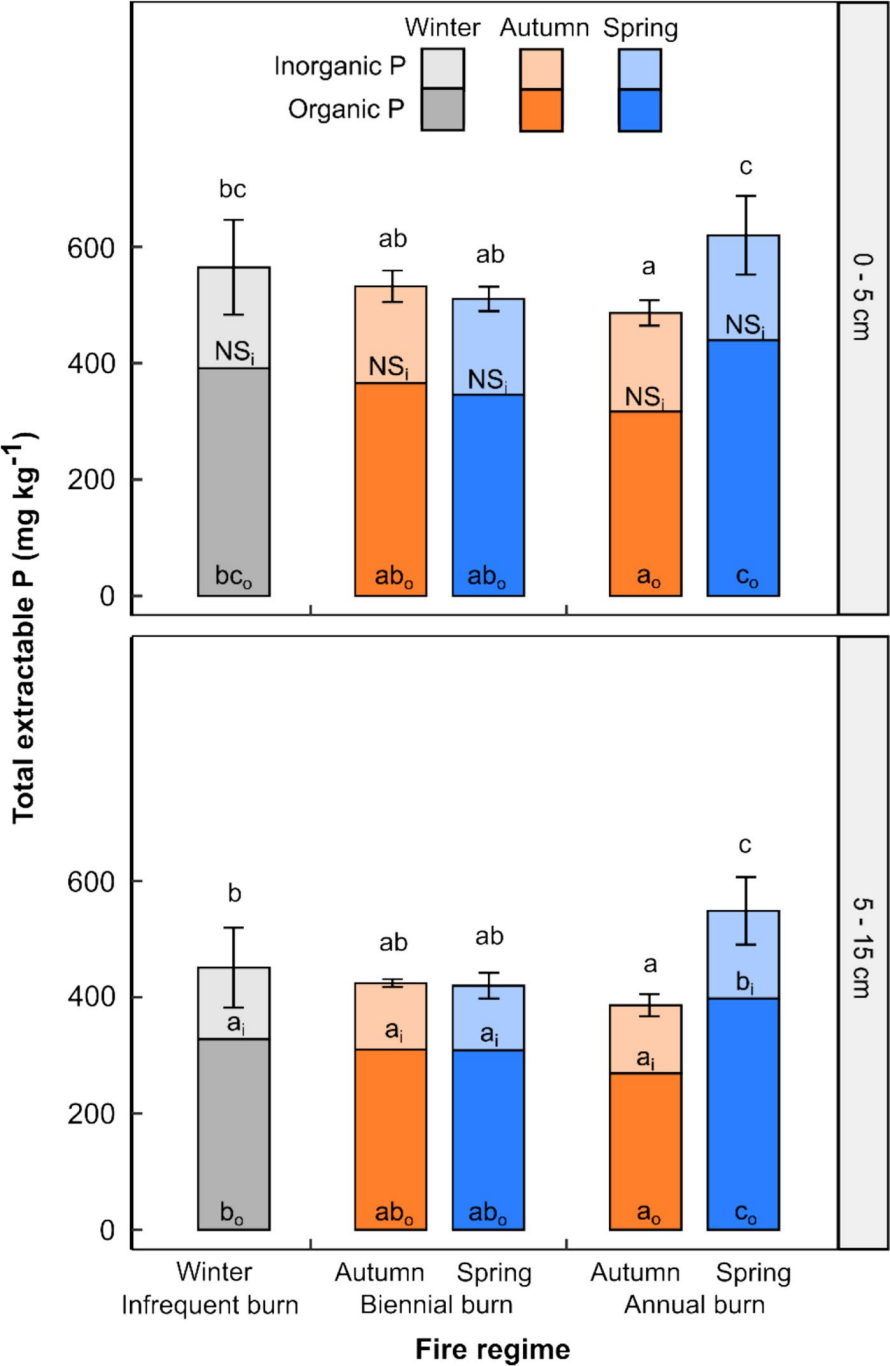
Organic and inorganic P (P_o_ and P_i_, respectively) as a fraction of NaOH-extractable soil P in the top 0–5 and 5–15 cm. The ANOVA for each fraction (NaOH-extractable P, P_o_ and P_i_) was run separately for each depth. Error bars reflect average NaOH-extractable P (± 1 SD, *n* = 3). Different letters represent significant treatment differences, per depth and for each fraction, determined by post hoc testing (Fisher’s protected LSD), at *α* = 0.05. Letters above each bar represent the comparison between treatments for NaOH-extractable soil P, while those with subscripts o and i reflect the comparison between treatments for P_o_ and P_i_, respectively

Significant differences in moderately labile soil P fractions, measured as NaOH-extractable P, were observed among annual burn treatments (Fig. 5). Annual spring burns resulted in higher concentrations of NaOH-extractable P compared to all other treatments, while annual autumn burns were associated with the lowest levels of NaOH-extractable P (*P* = 0.02, lsd_0.05_ = 72.48 for 0–5 cm and *P* = 0.003, lsd_0.05_ = 63.42 for 5–15 cm).

The inorganic fraction of NaOH-extractable P (P_i_) was largely unaffected by fire regime, except in the 5–15 cm layer, where concentrations were significantly higher under annual spring burns (*P* = 0.005, LSD_0.05_ = 17.61). In contrast, the organic fraction (P_o_)—which comprised an average of 69% of NaOH-extractable P in the 0–5 cm layer and 72% in the 5–15 cm layer—differed significantly among treatments at both depths (0–5 cm: *P* = 0.014, lsd_0.05_ = 60.70; 5–15 cm: *P* = 0.011, lsd_0.05_ = 58.67). Organic P concentrations were lowest under annual autumn burns and highest under annual spring burns (Fig. 5).

A small but significant difference in the P_o_:P_i_ ratio was observed with depth, with values of 2.18 in the 0–5 cm depth and 2.62 in the 5–15 cm depth (*P* < 0.001, lsd_0.05_ = 2.160). This increase in P_o_:P_i_ with depth was not affected by fire treatment (not shown).

The proportion of total P accounted for in the NaOH-extractable P fraction ranged from 60.69% to 79.68% in the 0–5 cm and 54.55% to 72.88% in the 5–15 cm depth (not shown).

**Fig. 6:**
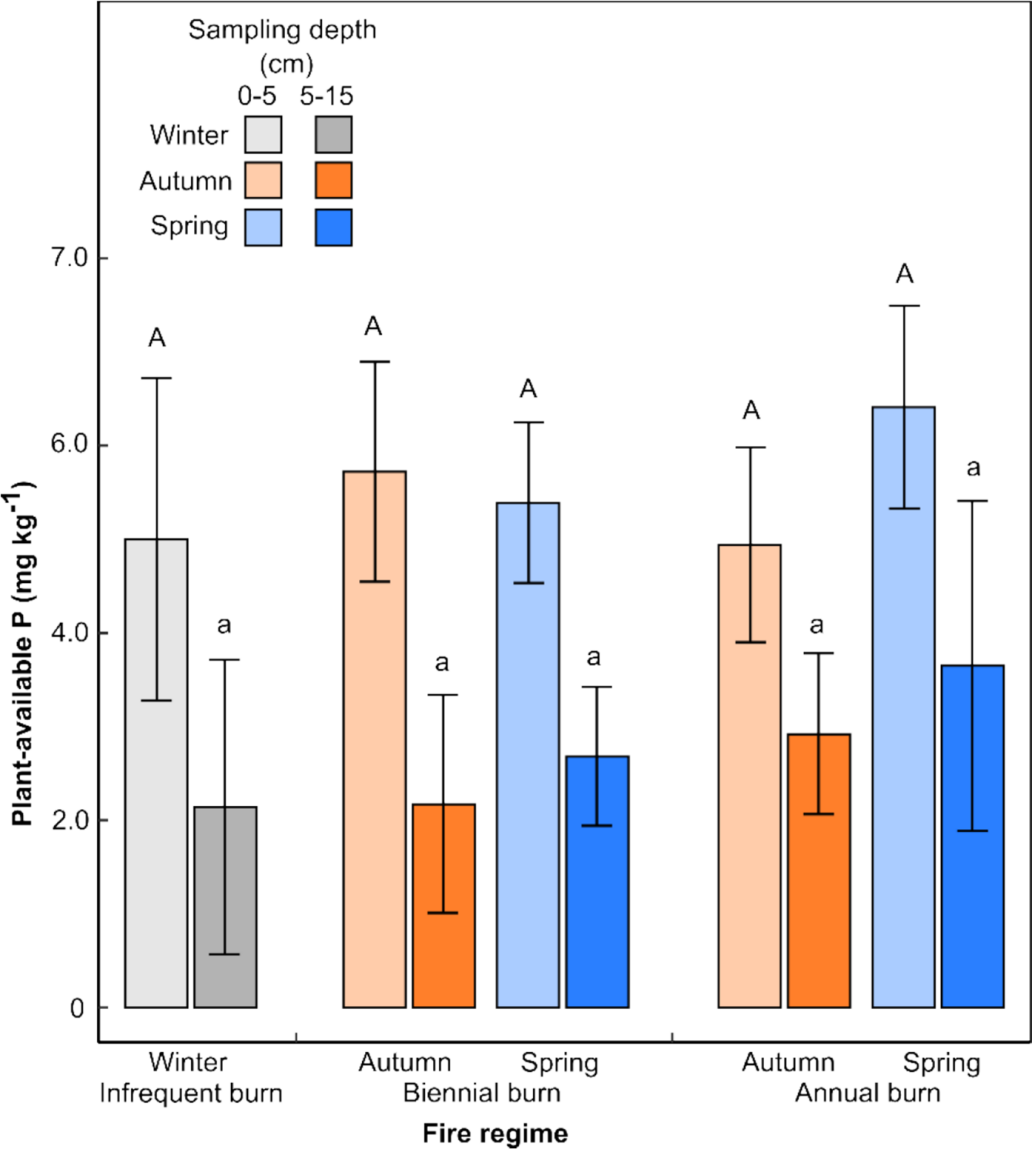
Mean plant-available soil P (± 1 SD, *n* = 3) for each treatment in the 0–5 and 5–15 cm sampling depths. Plant-available P declined with depth (*P* < 0.001, lsd_0.05_ = 0.765), but there were no statistically significant effects of the fire treatments at either sampling depth

Plant-available soil P was significantly lower (*P* < 0.001, lsd_0.05_ = 0.765) in the 5–15 cm sampling depth than in the top 0–5 cm (Fig. 6). Patterns in the response of plant-available P to fire regime appear similar to those observed for NaOH-extractable P, but differences were not statistically significant at either sampling depth (*P* = 0.475 and *P* = 0.445 for 0–5 and 5–15 cm depths, respectively).

## Discussion

We investigated the effects of fire frequency and season on topsoil P pools (total, organic, inorganic and plant-available) in the long-term Brotherton fire experiment in the montane grasslands of the uKhahlamba-Drakensberg, South Africa. We hypothesised that frequent burning would have little effect on total soil P but would shift its distribution, resulting in lower P_o_ and higher P_i_ and plant-available P. Total soil P ranged from 561 to 1053 mg kg^−1^. As predicted, total soil P was similar under infrequent and biennial burns; however annual spring burns unexpectedly produced higher values. Annual spring burns also showed higher P_o_ relative to P_i_, whereas seasonal differences were negligible in the biennial treatments. Despite these shifts in P_o_ and P_i_, plant-available P remained unchanged across all treatments, indicating a strong effect of fire season under high-frequency regimes on P_o_ and P_i_ partitioning, but no significant impact of fire on plant-available P.

Although highly weathered, Drakensberg topsoils maintain relatively high total P compared to other subtropical, highly weathered soils, but remain within the global range of 3–4087 mg kg^−1^ (He et al. 2021). These elevated values reflect the nutrient-rich basaltic parent material (Carbutt & Edwards 2015) and high soil organic matter content (Findlay et al. 2022) of the site. The persistence of high total P in these highly-weathered soils suggests a legacy of P retention, possibly linked to the amorphous mineral phases typical of Andosols that stabilise P through sorption and complexation, rather than ongoing replenishment or fire-induced changes. It remains unclear whether younger Drakensberg Andosols, with less-developed sorption capacity and greater contributions from primary mineral P, would exhibit even higher total P content.

While total P often constrains the potential sizes of major P pools (Hou et al. 2018), the key effect of fire in this system lies in its redistribution of P among organic and inorganic fractions. Previous studies have reported increases in soil P_i_ in P-limited systems subjected to repeated fire due to pyro-mineralisation of organic P (e.g., Schaller et al. 2015; Butler et al. 2018). In contrast, P_i_ in our study did not differ significantly among treatments in surface soils (0–5 cm) and was only modestly higher at depth (5–15 cm) under annual spring burns, suggesting that ash deposition and pyromineralisation play limited roles in P enrichment in these montane grasslands. Instead, the higher P_o_ concentrations observed under annual spring burns, particularly in the 5–15 cm layer (73% of NaOH-extractable P), likely reflect enhanced primary productivity (Everson & Everson 2016). At this depth, contributions from root biomass and turnover following fire may be especially important (Johnson & Matchett 2001; Wang et al. 2022; Luo et al. 2024), adding organic matter to the soil and accelerating nutrient turnover (Ojima et al. 1994; Johnson & Matchett 2001; Pathak et al. 2017). Increased root turnover may also enhance microbial demand for P, promoting immobilisation of P_i_ into microbial biomass (Huang et al. 2013), and further reinforcing P_o_ accumulation, although this mechanism was not directly measured here. The elevated P_o_:P_i_ ratios observed under annual burning support this interpretation, suggesting that P accumulation is driven more by organic inputs than by direct inorganic inputs from ash. Differences in reported P_o_ response to fire between this grassland and other grassy biomes likely reflect the distinct nature of fire regimes: fires in grasslands, particularly spring burns, typically have moderate severity, shorter durations and less complete combustion of aboveground biomass (Stavi 2019). These fires produce black ash, which retains partially combusted organic material (Pereira et al. 2014) and promote shifts toward greater P_o_. In contrast, prolonged burning of woody fuels in forests and savannas may ash even mineral-associated organic matter (Merino et al. 2019), potentially leading to greater P_i_ formation. Because organic matter is a major driver of P dynamics, patterns in P_o_ are expected to parallel those of C and N, which are also cycled through organic inputs (He et al. 2021). Previous work at Brotherton found higher concentrations of soil organic C and N under spring burns, consistent with enhanced belowground productivity and reduced ash losses (Findlay et al. 2022), which may support the observed accumulation of P_o_ in these plots.

**Fig. 7:**
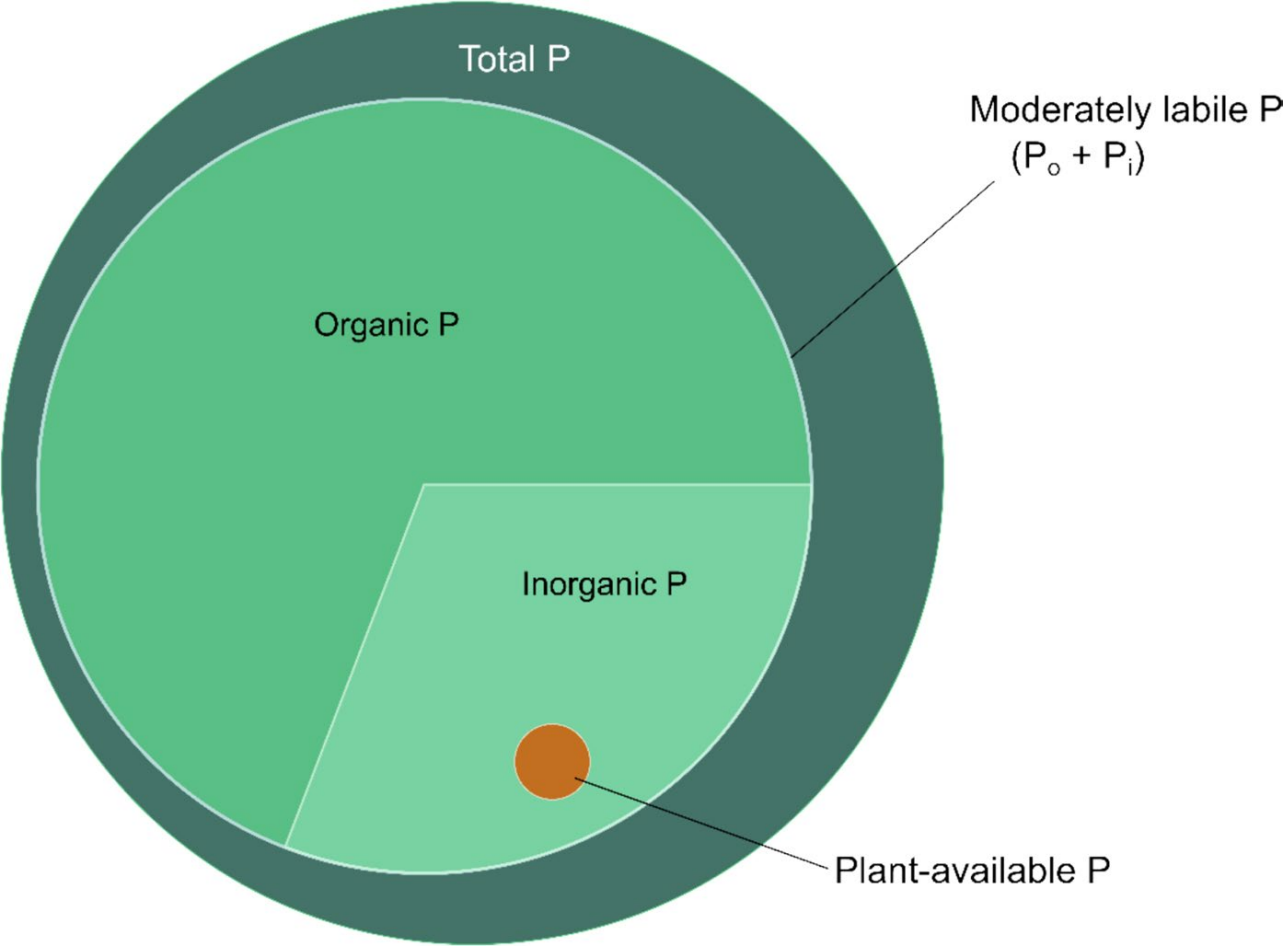
Proportional diagram showing the relative size of P pools in the top 0–5 cm of soil, averaged over all treatments. Moderately labile soil P (542.96 mg kg^−1^) averages 67% of the total ashed P (809.26 mg kg^−1^) and comprises P_o_ (371.96 mg kg^−1^; 69%) and P_i_ (171.01 mg kg^−1^; 31%). Plant-available *P* (5.45 mg kg^−1^) is included as contributing to P_i_ because it was determined using the Murphy & Riley (1962) method, which indicates P in an orthophosphate form (Blombäck et al. 2021)

Despite the high total P content, plant-available P (Ambic extraction) in this study remained low (Fig. 7). Interestingly, ongoing research suggests that grasses and forbs at this site are generally not severely P-limited (Findlay et al. 2025), and that plant species are able to maintain adequate P nutrition (at current levels of N-availability) despite low available P. This may reflect effective acquisition strategies such as high root surface area, enhanced phosphatase activity, carboxylate exudation, or associations with mycorrhizal fungi (Lambers et al. 2008); however, these mechanisms were not directly measured. The apparent disconnect between total and plant-available P points to a bottleneck in the P cycle, where increased organic P inputs do not translate to greater plant-available P. This decoupling likely arises from the combined effects of soil properties and climate on microbial activity, organic matter decomposition, and nutrient turnover. Seasonal shifts in climate can further modulate these processes, as observed by Hofstede (1995) in Colombian alpine Páramo grasslands, where wet-season decomposition was associated with increased P uptake despite lower plant-available soil P. A similar mechanism may help explain why vegetation at Brotherton maintains apparent P sufficiency during the growing season (Findlay et al. 2025), despite persistently low levels of plant-available P.

Relative to total P, however, the plant-available P remains low in these soils. Soils at Brotherton exhibit high P-retention capacity, with P-retention indices (Blakemore et al. 1987) averaging 91% in the 0–5 cm layer and 95% in the 5–15 cm soil layer (Supplementary Information Table S2), which is consistent with characteristics of Andosols (Shoji et al. 1993; Takahashi & Dahlgren 2016) and helps to explain why less than 4% of P_i_ is plant-available. In contrast to Butler et al. (2018), who reported that repeated burning increases P_i_ concentrations and P_i_:P_o_ ratios, effectively “resetting” soil P to a state characteristic of early-stage soils, P_o_:P_i_ ratios remain high with frequent spring fires at Brotherton and do not shift toward P_i_ dominance. Consequently, despite frequent burning, plant-available P remains constrained, reflecting both the inherent chemical properties of these Andosols and bottlenecks in microbial and mineral-mediated P cycling.

## Conclusions

Using data from the 39th year of a long-term fire experiment in the montane grasslands of the uKhahlamba-Drakensberg mountains, we found that fire regime significantly influences soil P pools. Specifically, annual spring burning resulted in higher P_o_ and, to a lesser extent, P_i_ relative to less frequent burns. Contrary to expectations, however, these differences did not translate into measurable changes in plant-available P. This apparent decoupling highlights a central finding: as found in other Andosols, P availability is constrained primarily by exceptionally high P-retention capacity (> 90% in both surface and subsoil layers), which limits P_i_ mobility and likely suppresses fire-driven increases in plant-available P. Our results also suggest seasonal modulation of fire effects, most evident in the contrast between annual spring and autumn burns, but these effects were not observed under less frequent burning, emphasizing the importance of both fire frequency and timing in shaping P cycling in montane grasslands.

Overall, while fire regime clearly influences the composition of soil P pools, the retention dynamics of alu-andic Andosols ultimately dominate P availability to plants, irrespective of fire-driven changes in inputs. These findings demonstrate the complex and sometimes counterintuitive effects of fire on nutrient dynamics in these unique montane grasslands.

## Supplementary Information

Below is the link to the electronic supplementary material.

Supplementary file1 (DOCX 38 KB)

## Data Availability

Soil phosphorus data supporting the findings of this study are not currently publicly available as they form part of the dataset for a PhD research project. All data will be archived on the South African Environmental Observation Network (SAEON) Open Data Platform (ODP) upon completion of the PhD. However, data are available from the corresponding author (Nicola Findlay) upon reasonable request.
