# Supplementary material for: Frequent fire alters soil total phosphorus but does not affect phosphorus availability in a montane grassland
Source: Biogeochemistry. 2026 Feb 10;169(2):13. doi: 10.1007/s10533-025-01304-w (PMC12953321; doi:10.1007/s10533-025-01304-w)
Supplement: Supplementary file 1 — Supplementary file1 (DOCX 38 KB) [file 10533_2025_1304_MOESM1_ESM.docx]

Supplementary information for

**Frequent fire alters soil total phosphorus but does not affect phosphorus availability in a montane grassland**

***Biogeochemistry***

Nicola J. Findlay^1,2*^, Guy Thibaud^2^, Alan D. Manson^3^, Paul J. Gordijn^4,5^, Max Rietkerk^1^, Martin J. Wassen^1^, Mariska te Beest^1,4,6^

*^1^Copernicus Institute of Sustainable Development, Utrecht University, Utrecht, The Netherlands*

*^2^Soil Fertility & Analytical Services, KwaZulu-Natal Department of Agriculture & Rural Development, Pietermaritzburg, South Africa*

*^3^School of Agricultural, Earth and Environmental Sciences, University of KwaZulu-Natal, Pietermaritzburg, South Africa*

*^4^South African Environmental Observation Network (SAEON), Grasslands, Forests and Wetlands Node, Pietermaritzburg, South Africa*

*^5^Biodiversity, Spatial Planning and Information, Ezemvelo KZN Wildlife, Pietermaritzburg, South Africa*

*^6^Centre for African Conservation Ecology, Nelson Mandela University, Port Elizabeth, South Africa*

*Correspondence to*: Nicola J. Findlay ([Nicky.Findlay@kzndard.gov.za](mailto:Nicky.Findlay@kzndard.gov.za))

**Introduction**

This supplementary document provides detailed statistical analyses supporting the results presented in the main manuscript. Table S1 contains ANOVA summaries corresponding to Figures 3, 4, and 5. Table S2 presents ANOVA results and treatment means for soil phosphorus retention, which are not presented in the results in the manuscript as they were *post hoc* analyses. These analyses enable transparency in the data interpretation and support the main findings on the effects of frequent fire on soil phosphorus, helping to contextualize the observed patterns described in the manuscript.

Table S1: Summary ANOVA tables for Manuscript Figures 3, 4 and 5

Table S2: Summary of ANOVA results and treatment means for P retention

**Table S1.** Summary of the ANOVA results for a) total ashed P, b) total extractable P, c) inorganic P, d) organic P and e) plant-available P, comparing the five fire treatments: infrequent burn (winter), biennial burn (autumn and spring) and annual burn (autumn and spring) at each sampling depth. Fisher’s protected LSD was used for *post hoc* testing when P<0.05.

Table S1. Continued on following page…/

**Table S1.** Continued

**Table S2.** Summary of ANOVA results and treatment means for P retention across the five fire treatments at each sampling depth. Fisher’s protected LSD was used for *post hoc* comparisons when P<0.05. Different letters after treatment means indicate significant treatment differences.
